# Hybrids of *Ixodes ricinus* and *Ixodes persulcatus* ticks effectively acquire and transmit tick-borne encephalitis virus

**DOI:** 10.3389/fcimb.2023.1104484

**Published:** 2023-01-20

**Authors:** Oxana A. Belova, Alexandra E. Polienko, Anastasia D. Averianova, Galina G. Karganova

**Affiliations:** ^1^ Laboratory of Biology of arboviruses, Federal State Autonomous Scientific Institution "Chumakov Federal Scientific Center for Research and Development of Immune-and- Biological Products of Russian Academy of Sciences" (Institute of Poliomyelitis), Moscow, Russia; ^2^ Department of Virology, Biological Faculty, Lomonosov Moscow State University, Moscow, Russia

**Keywords:** *Ixodes ricinus*, *Ixodes persulcatus*, hybrids, tick-borne encephalitis virus, vector, trans-stadial transmission

## Abstract

*Ixodes rici nus* and *Ixodes persulcatus* ticks are the main vectors of tick-borne encephalitis virus (TBEV), which has three main subtypes connected with certain tick species: the European subtype, associated with *I. ricinus*, and the Siberian and Far-Eastern subtypes, associated with *I. persulcatus*. Distribution ranges of these species overlap and form large sympatric areas in the East European Plain and Baltic countries. It has previously been shown that crossing of *I. ricinus* and *I. persulcatus* is possible, with the appearance of sterile hybrids. Hybridization of ticks can affect not only the spread of ticks but also the properties of natural foci of arbovirus infections, in particular TBEV. In the present study, we analyzed the effectiveness of virus transmission from infected mice to larvae and nymphs and trans-stadial transmission (from larvae to nymph and adult) in *I. ricinus*, *I. persulcatus*, and hybrids. For this purpose, we bred a hybrid generation from the crossing of *I. persulcatus* females and *I. ricinus* males, and we used the Siberian and European subtypes of TBEV. We showed that after feeding on infected mice, virus prevalence in engorged ticks decreased over time, and after molting, the opposite was true. In hybrids we observed the highest acquisition effectiveness and RNA copy numbers during Siberian TBEV subtype transmission. The efficiency of trans-stadial transmission of both TBEV subtypes was similar in hybrids and parental species. After the second trans-stadial TBEV transmission, a significant increase in ticks’ infection rates was observed only in specific subtype-tick combination. Our data demonstrate the possible features of TBEV circulation in the *I. ricinus* and *I. persulcatus* sympatry area.

## 1 Introduction

Tick-borne encephalitis (TBE) is a zoonotic vector-borne viral infection, the causative agent of which is the neurotropic tick-borne encephalitis virus (TBEV) of the genus *Flavivirus* (family *Flaviviridae*). Traditionally, three main subtypes of TBEV have been distinguished: European, Siberian, and Far Eastern (Ecker et al., 1999). During recent years, additional lineages and provisional subtypes have been reported: genotype 4 (TBEV-Bkl-1, strain 178–79) and genotype 5 (TBEV-Bkl-2, strain 886–84 and related East-Siberian isolates) (Demina et al., 2012), Himalayan subtype (TBEV-Him) (Dai et al., 2018) and Obskaya subtype (TBEV-Ob) (Deviatkin et al., 2020). The European subtype is common in Europe and the European part of Russia, while the Siberian and Far Eastern subtypes occupy a vast territory from Japan and the Russian Far East to the Baltic countries. The main vectors and long-term reservoirs of the virus in nature are *Ixodes persulcatus* in Asia and in some parts of Europe and *Ixodes ricinus* in Europe (Lindquist and Vapalahti, 2008). The epidemiology of TBEV is closely related to the ecological and biological characteristics of tick vectors, in particular their distribution ranges and periods of activity. Thus, the correspondence of the main TBEV subtypes with a certain species of the tick-vector is noted: the European subtype is associated with *I. ricinus* and the Siberian and Far Eastern subtypes are associated with *I. persulcatus*. It should be noted that the European and Siberian subtypes of TBEV often circulate in the same area in Eastern Europe and the European part of Russia (Lindquist and Vapalahti, 2008). At the same time, each of the subtypes retain evolutionary independence, presumably due to their narrow specialization to the tick organism.

Because of their long lifespan, ticks are not only TBEV vectors, but also the main reservoirs for the virus. In tick populations, the virus can be transmitted horizontally (from an infected tick to the vertebrate host and vice versa) and vertically (from an infected female to her progeny, also known as transovarial transmission) and trans-stadially (Nuttall and Labuda, 2003). During horizontal virus transmission, ticks can be infected with TBEV *via* feeding on an infected animal with sufficient level of viremia in the blood (viremic or systemic transmission) (Radda et al., 1969; Chunikhin and Kurenkov, 1979), or during cofeeding in close proximity with infected ticks on a host without viremia (non-viremic, non-systemic, or co-feeding transmission) (Alekseev and Chunikhin, 1990; Labuda et al., 1993). The high efficiency of the non-viremic transmission allowed researchers to draw conclusions about its crucial role in the maintenance of the TBEV in the natural endemic cycle (Randolph, 2011; Havlíková et al., 2013). Additionally, the possibility of sexual transmission was demonstrated, when TBEV was transmitted to females during copulation with infected males, the prospermia of which contained the virus (Chunikhin et al., 1983).

According to experimental data, the effectiveness of transovarial TBEV transmission varies greatly depending on the strain and dose of the virus, tick species, ticks’ infection route, and method of TBEV detection in ticks, and usually does not exceed 1% (Benda, 1958; Kondrashova and Filippovets, 1970). For continuous TBEV circulation in tick populations, trans-stadial virus transmission is essential. As with transovarial transmission, the effectiveness of trans-stadial transmission depends on many factors and varies from 0 to 100% (Naumov et al., 1980; Kurenkov et al., 1981; Danielová, 1990; Slovák et al., 2014; Migné et al., 2022). An assumption was made that in natural tick populations, the level of trans-stadial transmission is about 10–20% (Naumov et al., 1980).

Thus, the wide variety of TBEV transmission routes in natural foci guarantees continuous virus circulation and probably dictates the specificity of TBEV’s interrelation with a certain tick species. As a result, ticks determine the level of TBEV circulation in nature and affect the genetic and phenotypic properties of the viral population (Dzhivanyan et al., 1988; Labuda et al., 1994; Romanova et al., 2007; Růžek et al., 2008; Khasnatinov et al., 2009; Chausov et al., 2010; Belova et al., 2017). Thus, the evolution of TBEV has led to the formation of stable virus subtypes adapted to certain vector species and the whole eco-faunistic complex. However, the mechanisms and barriers that support the separation of subtypes, including in the sympatric area of the main vectors, remain unclear.

During recent years, significant changes have been recorded in the distribution of the main vectors of TBEV in Northern Europe, which in some cases led to changes in the eco-epidemiology of TBE and other tick-borne diseases (Tokarevich et al., 2011; Jaenson et al., 2012; Bugmyrin et al., 2013; Bugmyrin et al., 2014; Jaenson et al., 2016; Tokarevich et al., 2017; Soleng et al., 2018; Hvidsten et al., 2020; Vikse et al., 2020; Voyiatzaki et al., 2022). In Finland, in comparison with the previous national distribution map, compiled almost 60 years ago, the distribution border of *I. ricinus* and *I. persulcatus* shifted 200–300 km to the north, which led to the appearance of new foci of TBEV, as well as to an expansion of the sympatric zone of these two tick species (Laaksonen et al., 2017). In the sympatric zones of *I. ricinus* and *I. persulcatus*, a change in the main vector species for the Siberian and European TBEV subtypes is possible (Jääskeläinen et al., 2011; Jääskeläinen et al., 2016), which can affect the properties of the viral population. All this can lead to a change in the distribution and/or the emergence of new variants of TBEV and to a change in the epidemiological situation for TBE.

A separate issue is the existence of *I. ricinus* and *I. persulcatus* hybrids in sympatric zones and their influence on the properties of the virus. In the 1990s, the possibility of the existence of hybrid forms in the first generation was proved in laboratory experiments with reciprocal crossing of *I. ricinus* and *I. persulcatus*, while sterility of the obtained hybrids was noted (Balashov et al., 1998). Subsequently, we were able to repeat the experiments on obtaining hybrids of the indicated species of ticks, to study the morphology of the hybrids, and also to find hybrid individuals in nature (Bugmyrin et al., 2015; Bugmyrin et al., 2016), but their role as TBEV vectors has not been studied yet.

The problem of hybridization’s impact on vector competence has more often been explored for mosquitoes. For example, it is thought that hybrids between bird-biting *Culex pipiens* form *pipiens* and human-biting *Cx. pipiens* form *molestus*, which have an intermediate host preference, are ideal vectors to bridge West Nile virus (WNV) from birds to humans (Farajollahi et al., 2011; Fritz et al., 2015). In special experiments, it was shown that hybrids obtained from reciprocal crossings of two *Cx. pipiens* biotypes and *Cx. quinquefasciatus* were better WNV vectors than the parental species, as they showed higher infection and transmission rates (Ciota et al., 2013).

Thus, hybridization can affect the properties of natural foci of arbovirus infections; therefore, the study of hybrids of the main TBEV vectors seems highly relevant. Competence of the vector lies in its ability to acquire, preserve, and transmit the virus. In the present study, we compare the acquisition and transmission efficiency of the European and Siberian TBEV subtypes in *I. ricinus*, *I. persulcatus*, and their hybrids.

## 2 Materials and methods

### 2.1 Ticks and animals

Ticks were collected by flagging in allopatric territories in Russia: *I. ricinus* in the Voronezh (N 51.630165, E 39.671455) and Kaliningrad (N 55.159184, E 20.843275) regions, and *I. persulcatus* in the Republic of Karelia (N 62.063505, E 33.985511). From these ticks, laboratory colonies of *I. ricinus* and *I. persulcatus* were obtained and reared through one or two generations under controlled laboratory conditions. Original females were tested for the presence of TBEV by quantitative PCR and showed a negative result. In the laboratory, living ticks were kept in humidified glass tubes, as described previously (Belova et al., 2012). To maintain the laboratory colony, adult ticks were fed on rabbits (*Oryctolagus cuniculus*, breed “Soviet Chinchilla”, “Scientific Center of Biomedical Technology” RAS, branch “Stolbovaya”) under a cloth cap, as described previously (Belova et al., 2017). Larvae and nymphs were fed on laboratory mice (*Mus musculus*, outbred ICR mice, “Scientific Center of Biomedical Technology” RAS, branch “Stolbovaya”). Cardboard collars were put on the mice to prevent them from scratching the ticks, and then the mice were put in individual glass jars. After that, 100-150 larvae or 20-25 nymphs of *Ixodes* ticks were put onto the mice with a brush, the jars were covered with a nylon mesh to avoid spreading of ticks, and they were left for an hour for ticks to attach. Then, mice with attached ticks were transferred from the jars into individual cages with a slatted bottom and a water tray underneath. The water in the tray was changed daily and the engorged ticks that had fallen off were collected in humidified glass tubes. All animals were maintained according to the international guidelines for animal husbandry, including the recommendations of CIOMS, 1985 and the FELASA Working Group Report, 1996–1997. The study protocol was approved by the Ethics Committee of Chumakov FSC R&D IBP RAS (Institute of Poliomyelitis), protocol No 040321-1 from 04.03.2021.

To breed hybrids, in the first stage, virgin *I. persulcatus* females were obtained. The sex of ticks cultured in the laboratory was determined at the stage of engorged nymph (Balashov, 1972; Voltsit, 1986); large and small engorged nymphs were separated into different tubes, where they molted into males and females. In the next step, virgin *I. persulcatus* females were put into humidified glass tubes with *I. ricinus* males for several days, and then were fed on rabbits. To obtain adult hybrid ticks, immature hybrids were fed on laboratory mice. To avoid mating, female and male hybrids were separated into different glass tubes at the stage of engorged nymphs. Several *I. ricinus* and *I. persulcatus* specimens and obtained hybrids were analyzed genetically to confirm the ‘purity’ of the original species and the successful breeding of a hybrid generation (Litov et al., 2022).

### 2.2 Viruses and cells

In this work, two TBEV strains belonging to different subtypes were used:

− Strain EK-328 of the Siberian subtype, isolated from a pool of *I. persulcatus* ticks in 1972 in Estonia (GenBank ID DQ486861) (Romanova et al., 2007), with a passage history of 12 passages through mice brain (outbred ICR mice, 6-7 g) and 1 passage in the pig embryo kidney (PEK) cell line;
− Strain LK-138 of the European subtype, isolated from a pool of *I. ricinus* ticks in 1972 in Lithuania (GenBank ID GU125720) (Kozlovskaya et al., 2010), with a passage history of two passages through suckling mice brain (outbred ICR mice) and four passages in the PEK cell line.

Additionally, strain Sabin, poliovirus type I was used as an internal control during RNA isolation. Viruses were used as culture supernatants from the infected PEK cell line. A PEK cell line (Institute collection) was maintained at 37°C in Medium 199 (Chumakov FSC R&D IBP RAS, Russia), supplemented with 5% fetal bovine serum (FBS, Gibco). Virus production and cell culture maintenance have been described previously (Romanova et al., 2007).

### 2.3 50% plaque reduction neutralization test

Plaque reduction neutralization test (PRNT50) was performed as described earlier (Tuchynskaya et al., 2021) on PEK cell monolayers in 24-well plates. Mouse blood samples were collected *via* decapitation; serum was separated from the clots by centrifugation and stored in aliquots at -20°C. For the test, 3-fold dilutions of sera from individual mice in medium 199 with 2% FBS (Gibco) were incubated with TBEV samples (1:1, v:v) in the concentration of 30–40 plaque forming unit per well for 1 h at 37°C. Virus-serum mixture (100µL) was added to PEK cells in 24-well plates, incubated for 1 h at 37°C in a CO_2_-incubator, coated with 1.26% methylcellulose (Sigma, St Louis, MO, USA) in medium 199 with Hanks and Earle’s salts (2:1, v:v) and 2% FBS, and left for incubation for 6 days at 37°C in a CO_2_-incubator. Then the cells were fixed with 96% ethanol and stained with 0.4% gentian violet dye. Every experiment included controls, i.e., negative and positive murine sera with known antibody titers. The neutralizing antibodies (NAb) titers were calculated according to the modified Reed and Muench method (Reed and Muench, 1938).

### 2.4 Detection of TBEV by quantitative PCR

RNA was isolated using TRI reagent (Sigma-Aldrich, USA) according to the manufacturer’s protocol. As an internal control, a known amount (10^5^ RNA copies) of the attenuated type I Sabin poliovirus vaccine strain was added to each sample before extraction. Reverse transcription was carried out with primers specific for TBEV and poliovirus (**Table S1**) and M-MLV reverse transcriptase (Promega, Madison, WI), according to the manufacturer’s protocol.

Quantitative PCR (qPCR) was carried out using DNA Engine Analyser (BioRad, USA) with a qPCR kit (Syntol, Russia) and specific primers to TBEV and poliovirus (**Table S1**), as described previously (Tuchynskaya et al., 2021). Viral RNA quantity in the samples was expressed as a decimal logarithm of the genome copy number (GCN) per sample (log_10_GCN/sample), i.e. per tick, pool of ticks or per 1 mL of 10% mouse brain suspension. The sample was considered as positive for the presence of TBEV if the number of GCN in the PCR reaction was more than 100 copies. For experimental groups, the geometric mean number of RNA copies (GMNC) was calculated considering only positive samples.

### 2.5 Sample preparation

Ticks were homogenized in saline solution (0.9% NaCl) (Chumakov FSC R&D IBP RAS, Russia) using the laboratory homogenizer TissueLyser II (QIAGEN, Germany). Engorged larvae and questing nymphs were homogenized in pools of three individuals, and engorged nymphs and adults were homogenized individually in 300 µL of saline solution. Brain samples of sacrificed mice were weighed and manually homogenized in saline solution to obtain 10% suspensions, which were aliquoted and stored at -80°С.

### 2.6 Experiments with TBEV transmission

**Figure 1:**
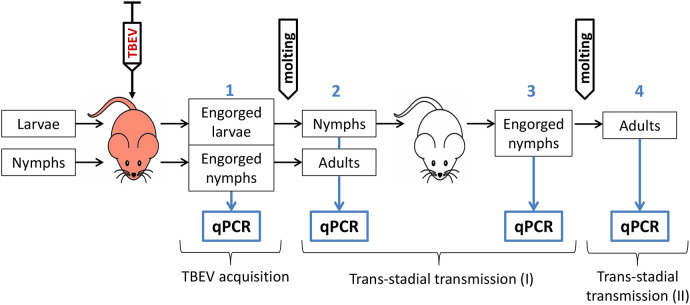
Schematic illustration of the experiment regarding TBEV transmission in *Ixodes ricinus, Ixodes persulcatus* ticks and their hybrids. Red mouse—TBEV-infected mice, white mouse—uninfected mice, qPCR—quantitative PCR. Blue numbers indicate different stages of the experiment.

The scheme of the experiment is shown in **Figure 1**. To model the natural route of the ticks’ infection with TBEV, larvae and nymphs of *I. ricinus* and *I. persulcatus* and larvae of hybrids, obtained from the crossing of *I. persulcatus* females and *I. ricinus* males, were fed on TBEV-infected laboratory mice. Mice (18–20 g) were infected *via* intraperitoneal injection of 0.3 mL of a virus-containing culture supernatant of the strain EK-328 (Siberian subtype, infection dose – 7.0 log_10_PFU) or strain LK-138 (European subtype, infection dose – 5.8 log_10_PFU). After one hour post infection (p.i.), larvae and nymphs were put on mice for feeding. After engorgement, one-third of the ticks were frozen at -80°С for analysis of the effectiveness of TBEV acquisition, and the rest were kept in humidified tubes for molting to study the trans-stadial transmission of the virus. After 9 to 31 days post molting, all adult ticks and half of the nymphs were frozen. The rest of the nymphs were fed on uninfected mice to assess the possibility of second trans-stadial TBEV transmission. After 7-14 days post ticks feeding (d.p.f.), mice were decapitated and blood and brain samples were harvested. Some of the engorged nymphs were frozen 30–71 d.p.f., and the rest were frozen 30–149 days post molting to adults. All ticks were analyzed for the presence of TBEV by qPCR.

### 2.7 Statistical analysis

Pooled prevalence was calculated using a maximum likelihood estimator with the assumption of 100% test sensitivity and specificity (Williams and Moffitt, 2001). The calculations were performed on the EPITOOLS web platform (Sergeant, 2018). In cases, when all the analyzed pools were positive, then the LaPlace adjustment method was used (confidence interval calculator for a completion rate). Statistical analyses were performed using OriginPro 8 SR4 (v8.0951, Northampton, MA, USA). To compare TBEV prevalence between groups, Fisher’s exact test or chi-square test were used. For other data, the significance of differences between groups was estimated according to the Mann–Whitney U-test (for two groups) and the Kruskal–Wallis H-test (for three groups). For multiple comparisons correction, Bonferroni correction was applied.

## 3 Results

### 3.1 TBEV transmission from infected mice to immature ticks

**Figure 2:**
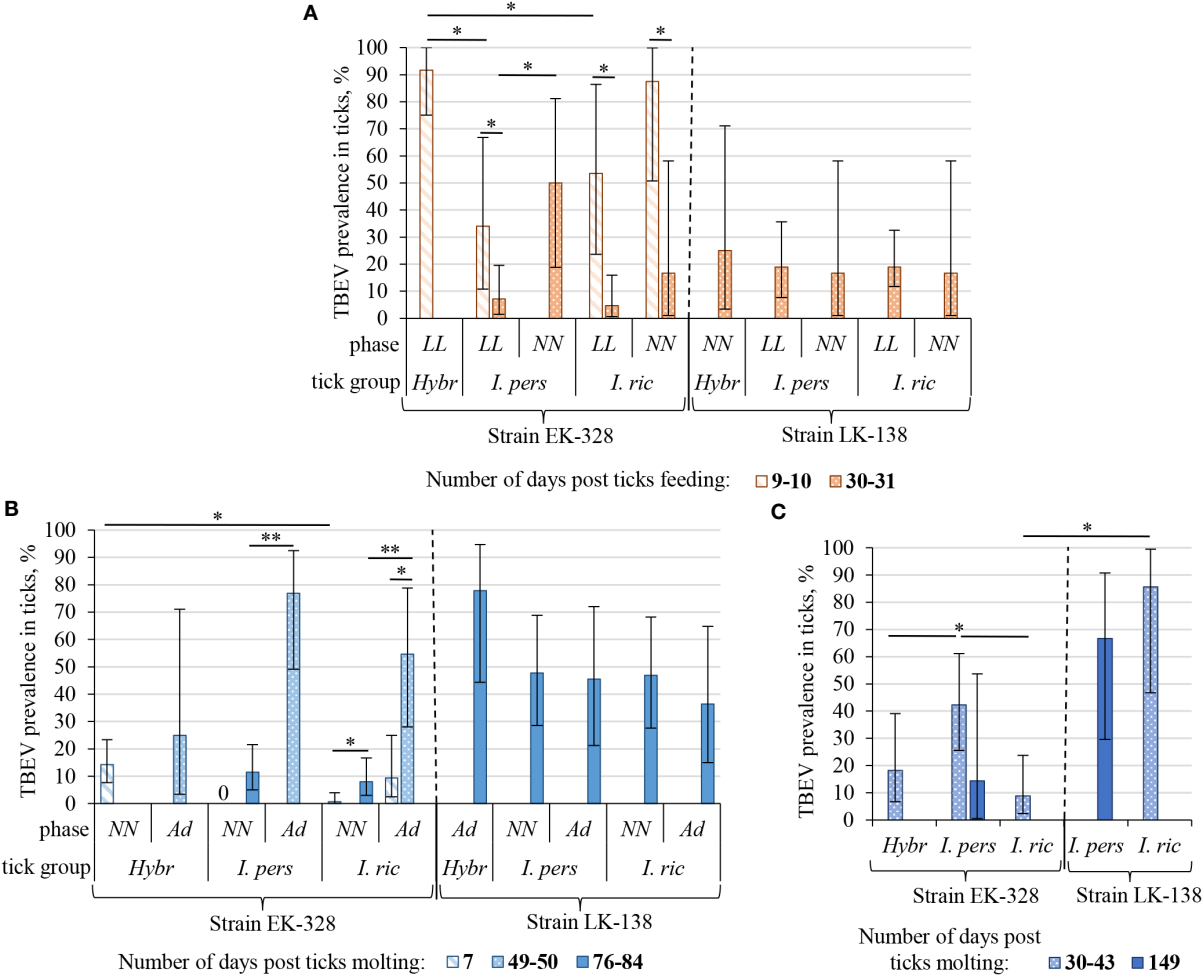
Prevalence of TBEV in *I. ricinus* (*I. ric*), *I. persulcatus* (*I. pers*), and hybrid (*Hybr*) ticks at different period after feeding/molting: **(A)** in engorged larvae (LL) and nymphs (NN) after feeding on infected mice; **(B)** in questing nymphs (NN) and adults (Ad) after first molting; **(C)** in adults after second molting. Data for the strains EK-328 and LK-138 are separated with dashed line. Engorged larvae and questing nymphs were studied in pools of three, others – individually, TBEV prevalence in a pool of ticks was calculated using a maximum likelihood estimator with the assumption of 100% test sensitivity and specificity on the EPITOOLS web platform (Sergeant, 2018). Whiskers show 95% confidence interval of the infection rate; significant differences between groups according to Fisher’s exact test/chi-square test: * p<0.05, ** p<0.001.

**Figure 3:**
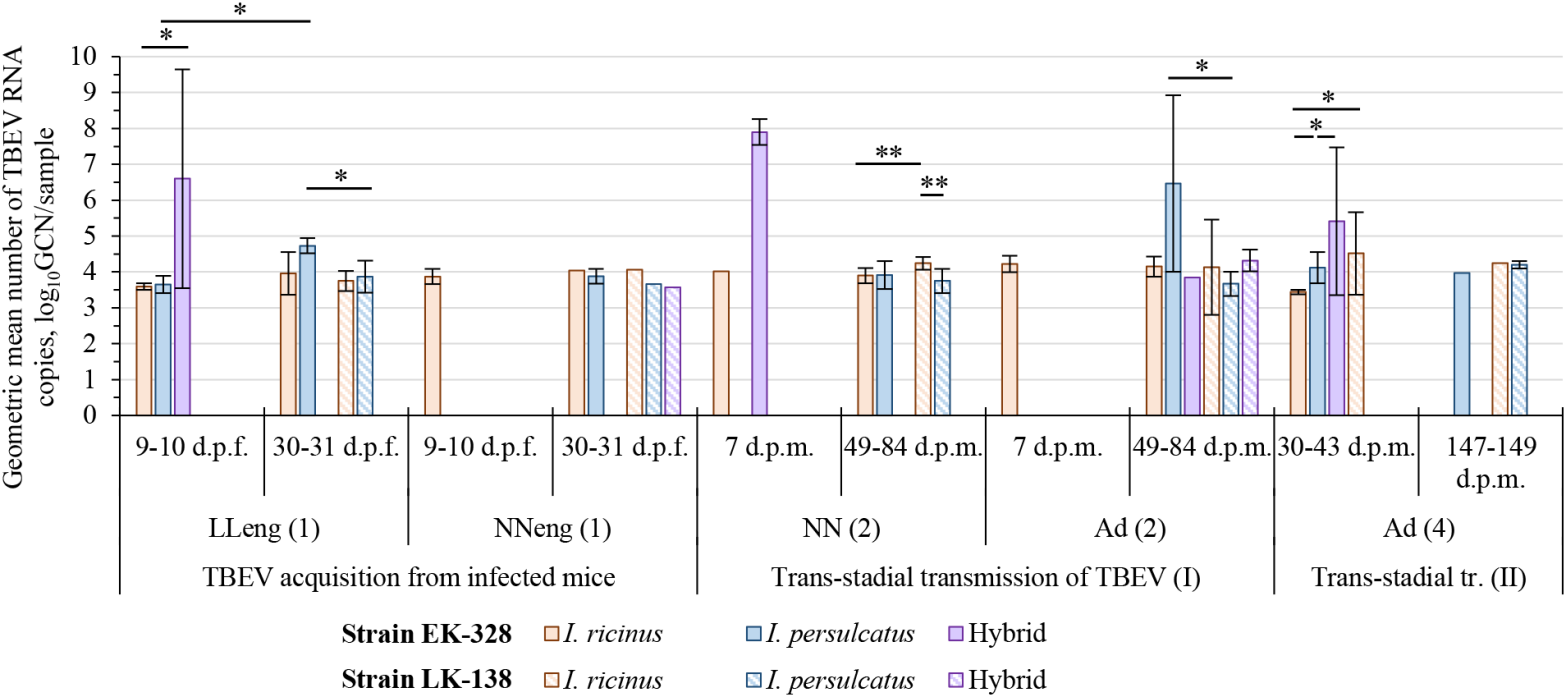
Geometric mean number of TBEV RNA copies in tick samples at different period after feeding/molting. Engorged larvae (LL_eng_ (1)) and questing nymphs (NN (2)) were studied in pools of three individuals, engorged nymphs (NN_eng_ (1)) and questing adults (Ad (2), Ad (4)) were analyzed individually; the number of TBEV RNA copies is shown for sample (a pool or individual tick). Whiskers show standard deviation; significant differences between groups according to Mann–Whitney U-test (2 groups) or Kruskal-Wallis H-test (3 groups): * p<0.05, ** p<0.001; d.p.f.—days post feeding on TBEV infected mice; d.p.m.—days post molting.

In this experiment two TBEV strains were used – EK-328 of the Siberian subtype, and LK-138 of the European subtype. The results are shown in **Table 1**; **Figure 2** and **Table S2**. The geometric mean amount of TBEV RNA for different tick groups are shown in **Figure 3** and **Table S3**.

**Table 1:** Effectiveness of the Siberian (strain EK-328) and European (strain LK-138) TBEV subtypes’ transmission in *Ixodes ricinus*, *Ixodes persulcatus* and hybrid ticks at different period after feeding/molting.

TBEV strain	Tick group	TBEV acquisition from infected mice			Trans-stadial transmission of TBEV (I)				Trans-stadial tr. (II)	
		LL_eng_* (1^#^)		NN_eng_ (1)	NN* (2)		NN_eng_ (3)	Ad (2)	Ad (4)	
		9-10 d.p.f.	30-31 d.p.f.	30-31 d.p.f.	7 d.p.m.	49-84 d.p.m.	129-137 d.p.m. (30-32 d.p.f.)	49-84 d.p.m.	30-43 d.p.m.	147-149 d.p.m.
EK-328	*I.ric*	53.6% **^a,c^** [23.7-86.4] (16/30)	4.7% **^c^** [0.6-15.9] (2/45)	16.7% [1.1-58.2] (1/6)	0.7% **^f,g^** [0.0-4.4] (1/138)	8.0% **^g,h,j^** [3.0-16.7] (6/81)	21.1% [8.0-43.9] (4/19)	54.6% **^j^** [28.0-78.8] (6/11)	8.8% **^m,n^** [3.2-29.8] (3/34)	nd
	*I.pers*	34.1% **^b,d^** [10.8-66.8] (7/21)	7.2% **^d,e^** [1.5-19.6] (3/45)	50% **^e^** [18.8-81.2] (3/6)	0% [0.0-13.5] (0/21)	11.5% **^i,k,o^** [6.0-20.7] (9/78)	10.0% [0.0-42.6] (1/10)	76.9% **^k^** [49.1-92.5] (10/13)	42.3% **^m,o^** [25.5-61.1] (8/14)	14.3% [0.5-53.4] (1/7)
	Hybrid	91.7% **^a,b^** [75.1-100] (27/30)	nd	nd	14.3% **^f^** [7.7-23.4] (15/105)	nd	nd	25% **^l^** [3.4-71.1] (1/4)	18.2% **^m^** [6.7-39.1] (4/22)	nd
LK-138	*I.ric*	nd	9.8% [0.7-19.0] (4/45)	16.7% [1.1-58.2] (1/6)	nd	46.9% **^h^** [27.6-68.2] (28/60)	37.5% [13.5-69.6] (3/8)	36.4% [15.0-64.8] (4/11)	85.7% **^n^** [46.7-99.5] (6/7)	1/1
	*I.pers*	nd	18.9% [6.1-31.7] (9/45)	16.7% [1.1-58.2] (1/6)	nd	47.7% **^i^** [29.5-66.0] (30/63)	25.0% [6.3-60.0] (2/8)	45.5% [21.3-72.0] (5/11)	nd	66.7% [29.6-90.8] (4/6)
	Hybrid	nd	nd	25% [3.4-71.1] (1/4)	nd	nd	nd	80.0% **^l^** [36.0-98.0] (4/5)	nd	nd

Infection rates of ticks with TBEV determined by qPCR are presented: percentage of positive ticks [CI 95%] (number of positive ticks/total analyzed). *I.ric, Ixodes ricinus; I.pers, Ixodes persulcatus*, Hybrid, ticks obtained from the mating of *I. persulcatus* females with *I. ricinus* males; nd, no data; LL_eng_, engorged larvae; NN_eng_, engorged nymphs; NN, questing nymphs; Ad, adult ticks; d.p.f., days post feeding; d.p.m., days post molting; *—engorged larvae and questing nymphs were studied in pools of three, TBEV prevalence was calculated using a maximum likelihood estimator with the assumption of 100% test sensitivity and specificity on the EPITOOLS web platform (Sergeant, 2018); ^#^—numbers indicate different stages of the experiment according to the **Figure 1**; superscript letters indicate statistically significant differences between groups according to Fisher’s exact test/chi-square test (p ≤ 0.01), groups with the same letter are significantly different from each other.

Since we assumed the possible influence of the physiological state of ticks on their infection rate (IR), ticks were analyzed in different periods after feeding on infected mice, and, subsequently, at different periods after molting (**Figures 2**, **3**; **Table 1**, **S2**). After complete feeding on infected mice, the analysis of ticks regarding the presence of TBEV was carried out after 9–31 days. Longer incubation of larvae and nymphs after feeding led to a decrease in the strain EK-328 prevalence in tick suspensions (**Figure 2A**; **Table 1**, **S2**). Despite the IR decrease at a later time after feeding, the number of RNA copies of the strain EK-328 in engorged *I. persulcatus* larvae was higher at 30 d.p.f. than after 9 days (**Figure 3**; **Table S3**).

The efficiency of the acquisition of the strain EK-328 by hybrid larvae was significantly higher than that of *I. ricinus* and *I. persulcatus*, reaching 91.7% at 9 d.p.f. (Chi-square test, p<0.01, **Table 1**; **Figure 2A**). Additionally, hybrids differed from parental species by a higher number of RNA copies of the strain EK-328; differences were significant between hybrids and *I. ricinus* (Mann–Whitney test, p<0.05, **Figure 3**; **Table S3**). The IR of *I. ricinus* and *I. persulcatus* larvae with the strain LK-138 was determined on the 31st d.p.f., was low and did not differ from IR with the strain EK-328 (**Table 1**, **S2**; **Figure 2A**). It should be noted that in the engorged *I. persulcatus* larvae analyzed on the 30th d.p.f., the number of RNA copies of the strain EK-328 was higher than that of the strain LK-138 (Mann–Whitney test, p<0.05, **Figure 3**; **Table S3**).

The effectiveness of the acquisition of the strain EK-328 by *I. ricinus* and *I. persulcatus* nymphs was higher than by the larvae, but the differences were significant only for *I. persulcatus* (Fisher exact test, p<0.05, **Table 1**, **S2**). The proportion of nymphs of the two tick species and their hybrids infected with the strain LK-138 was determined on the 30th d.p.f. and was low, similar to that for larvae (**Figure 2**; **Table 1**, **S2**).

Thus, the highest efficiency of acquisition of the strain EK-328 from infected mice was observed in hybrid larvae, in which the level of virus reproduction was 2–3 log_10_GCN higher than in the parental tick species.

### 3.2 Trans-stadial TBEV transmission

In the present study, we had two stages of trans-stadial TBEV transmission (**Figure 1**). In the first stage, larvae and nymphs, which fed on TBEV-infected mice, molted to the next developmental stage (nymphs and adults, respectively). Questing nymphs further fed on uninfected mice and molted to adults, which was the second trans-stadial transmission.

Ticks were analyzed for the presence of TBEV at different days post molting (d.p.m.), and in some cases there was a difference in the ticks’ IRs (**Figure 2B**; **Table 1**, **S2**). After the first molting, ticks were analyzed after 7–84 d.p.m. A longer period after molting favored a higher percentage of TBEV-positive ticks. Significant differences were obtained in the analysis of questing *I. ricinus* nymphs [Fisher exact test, p<0.01, **Figure 2B**; **Table 1**, **S2**, NN(2)] and adults [Chi square test, p<0.01, **Figure 2B**; **Table S2**, Ad(2)] for the presence of the strain EK-328 at 7 and 49–50 d.p.m. At the same time, there was no significant difference in the number of copies of TBEV RNA in ticks at different time points (**Figure 3**; **Table S2**). After second trans-stadial transmission (nymphs→adults), adult ticks were analyzed for the presence of TBEV 5–149 d.p.m. [**Table S2**, Ad(4)]. We did not find any dependence of TBEV prevalence on the number of days after second molting, as was observed during the virus acquisition and the first trans-stadial transmission (**Figure 2C**). However, it is worth noting that, even after 147-149 d.p.m. (332 days after feeding on infected mice and 194 days after feeding on uninfected mice), both strains were detected in ticks (**Figure 2C**; **Table 1**, **S2**) and the number of TBEV RNA copies was quite high (**Figure 3**; **Table S3**).

#### 3.2.1 First trans-stadial TBEV transmission

The effectiveness of the first trans-stadial transmission of the two TBEV strains was different. The maximal prevalence of the strain EK-328 in molted larvae of all three tick groups was rather low and did not exceed 15% [**Figure 2B**; **Table 1**, **S2**, NN(2)]. The highest IR was observed in hybrids (14.3%) (determined at 7 d.p.m.), which significantly differs from IR of *I. ricinus* (Chi square test, p<0.01), and *I. persulcatus* IR at this time period was 0% (**Table 1**; **Figure 2B**). The number of RNA copies of the strain EK-328 in positive hybrids was quite high, but could not be compared with other tick groups due to lack of data at 7 d.p.m. The IR of *I. ricinus* and *I. persulcatus* molted larvae with the strain LK-138 reached almost 50% (determined at 49-84 d.p.m.) and was significantly higher than that with EK-328 [**Table 1**, NN(2)]. The number of RNA copies of the strain LK-138 was significantly higher in *I. ricinus* molted larvae than in *I. persulcatus* (Mann–Whitney test, p<0.001, **Figure 3**; **Table S2**, **S3**). In addition, in *I. ricinus*, the number of RNA copies of the strain LK-138 was higher than that of the strain EK-328 (Mann–Whitney test, p<0.001, **Figure 3**; **Table S2**, **S3**). Feeding of questing *I. ricinus* and *I. persulcatus* nymphs after the molting of larvae slightly increased the prevalence of the strain EK-328 in *I. ricinus* [**Table 1**, **S2**, NN_eng_(3)] and increased the prevalence and number of LK-138 RNA copies in *I. persulcatus* [**Table S2**, NN_eng_(3), 221 d.p.m], but the differences were insignificant.

The prevalence of the strain EK-328 in molted nymphs of *I. ricinus* and *I. persulcatus* at 49-84 d.p.m. was significantly higher than that in molted larvae [Chi square test, p<0.001, **Figure 2B**; **Table 1**, Ad(2)]. The highest IR with the strain EK-328 was observed in *I. persulcatus* molted nymphs (**Table 1**; **Figure 2B**), but the difference was insignificant. Additionally, the highest number of RNA copies of the strain EK-328 was observed in *I. persulcatus* [**Figure 3**; **Table S2**, **S3**, Ad(2)], but the difference was not statistically significant. Prevalence of the strain LK-138 in *I. ricinus* and *I. persulcatus* molted nymphs was 36.4% and 45.5%, respectively, which was similar to that in molted larvae, and did not differ from the prevalence of the strain EK-328 in these tick species. In molted hybrid nymphs, the prevalence of the strain LK-328 was the highest, and significantly differed from EK-328’s prevalence [Chi square test, p<0.05, **Table 1**, Ad(2)]. In molted *I. persulcatus* nymphs, the number of RNA copies of the strain EK-328 was significantly higher than that of the strain LK-138 (Mann–Whitney test, p<0.01, **Figure 3**; **Table S2**, **S3**).

To estimate the trans-stadial TBEV survival rate, we need to divide the ticks’ IR after trans-stadial transmission by the effectiveness of the virus acquisition from infected mice. Unfortunately, we could estimate the trans-stadial survival rate only for the strain EK-328, since the efficiency of LK-138’s acquisition by ticks from infected mice was lower than the efficiency of trans-stadial transmission. Therefore, the trans-stadial survival rate of the strain EK-328 in larvae of *I. ricinus* and *I. persulcatus* and hybrids was 14.9%, 33.7%, and 15.6%, respectively. For hybrids, this indicator is most likely underestimated, since the efficiency of trans-stadial transmission was determined at 7 d.p.m. and could be higher at the later time period.

Thus, there were almost no significant differences in the efficiency of the trans-stadial transmission of both strains between the different groups of ticks. At the same time, in the *I. persulcatus* molted nymphs (Ad(2)) infected with the strain EK-328 and in hybrid molted nymphs infected with the strain LK-138, the IR and the number of TBEV RNA copies were slightly higher than in the other groups of ticks. Differences in the efficiency of trans-stadial transmission between the two strains were significant for *I. ricinus* and *I. persulcatus* molted larvae [NN(2)) and for hybrid molted nymphs (Ad(2)].

#### 3.2.2 Second trans-stadial TBEV transmission

After the second trans-stadial transmission, the IRs of adult ticks with the strain EK-328 were generally lower than those of adult ticks with the strain LK-138 [**Figure 2C**; **Table 1**, **S2**, Ad(4)]. The differences were significant for *I. ricinus*, the IR of which with the strain LK-138 was almost 10 times higher than with the strain EK-328 at 30-43 d.p.m. Chi-square test, p<0.01, **Figure 2C**; **Table 1**, **S2**). Additionally, in *I. ricinus*, the number of RNA copies of the strain LK-138 was significantly higher than that of the strain EK-328 (Mann–Whitney test, p<0.01, **Figure 3**; **Table S3**). The highest EK-328 prevalence was observed in *I. persulcatus* [Chi-square test, p<0.05), and when compared to the first trans-stadial transmission rate (NN(2)], this value increased by 3.7 times (Chi- square test, p<0.001, **Table 1**). Interestingly, the highest number of EK-328 RNA copies was retained in hybrids after the second trans-stadial transmission (Kruskal-Wallis test, p<0.01, **Figure 3**; **Table S3**). The IRs of adult ticks with the strain LK-138 was high and did not differ between *I. ricinus* and *I. persulcatus*. However, the IR of *I. ricinus* after the second trans-stadial transmission [Ad(4)) increased by almost two times compared to the first trans-stadial transmission (NN(2)], and the difference was statistically significant (Chi square test, p<0.05, **Table 1**).

### 3.3 Transmission of TBEV from molted larvae to laboratory mice

Questing *I. ricinus, I. persulcatus*, and hybrid nymphs, obtained after molting of TBEV infected larvae, were fed on uninfected ICR mice, and after 7-14 days post ticks feeding mice were decapitated and blood and brain were harvested. Results of the sera (PRNT50) and brain suspensions (qPCR) analysis are shown in the **Table 2**. It should be noted that mice showed no or mild symptoms of the disease. The latter could also be associated with blood loss due to tick feeding. According to our data, 78.3% (18/23) of mice had NAb titer in the sera higher than 1 log_10_ and/or a detectable level of TBEV RNA in the brain. There were no differences in efficiency of TBEV transmission and in number of TBEV RNA copies in mice brains either between tick groups or between virus strains. Thus, despite the small sample size, we were able to show the ability of hybrid nymphs to transmit both TBEV strains to mice.

**Table 2:** Effectiveness of the Siberian (strain EK-328) and European (strain LK-138) TBEV subtypes transmission from infected *Ixodes ricinus*, *Ixodes persulcatus*, and hybrid questing nymphs (infected as larvae) to laboratory mice.

Nymphs group	TBEV strain	Number of mice with NAb titer against TBEV ≥1 log_10/_total tested	TBEV RNA level in mice brains		Total number of infected mice*
			Number of positive mice/total tested	Mean RNA copy number, log_10_GCN/mL	
Hybrid	EK-328	1/2	1/2	4.45	1/2
	LK-138	1/1	1/1	4.60	1/1
*I. ricinus*	EK-328	3/6	4/6	4.36 ± 0.21	4/6
	LK-138	1/3	3/3	4.54 ± 0.27	3/3
*I. persulcatus*	EK-328	4/6	4/6	4.43 ± 0.40	4/6
	LK-138	3/5	4/5	4.55 ± 0.10	5/5

NAb—neutralizing antibodies; log_10_GCN/mL—decimal logarithm of the genome copy number in mL of 10% brain suspension; * the mouse was considered infected if the NAb titer against TBEV in the blood serum was ≥1 log_10_ and/or a detectable level of TBEV RNA in the brain was observed.

## 4 Discussion

In this work, we investigated the features of transmission of the European and Siberian TBEV subtypes in the main vectors and reservoirs—*I. ricinus* and *I. persulcatus* ticks, and their hybrids. Two strains were selected for the experiments, which were isolated from possibly sympatric areas of *I. ricinus* and *I. persulcatus.* It should be noted that the number of nymphs used and analyzed in our work was much lower than the number of larvae, which is associated with the peculiarities of ticks’ maintenance in laboratory conditions and limited resources. Despite this, important statistically significant results were obtained in the work. We expected that with a specific tick–strain combination, we would see a faster adaptation of the virus to the tick organism, which would possibly manifest itself in the form of more intensive TBEV reproduction or in the more efficient transmission of the virus. For all ticks, the number of RNA copies was determined by the qPCR method, since, in our opinion, this is the most accurate method for determining the concentration of the virus in the sample. In most studies of TBEV transmission, the infection of ticks with the virus was assessed by the amount of infectious virus determined by the plaque assay in a mammalian cell line (Labuda et al., 1993; Slovák et al., 2014; Khasnatinov et al., 2016), *via* intracerebral injection into suckling mice brains (Mishaeva and Erofeeva, 1979; Danielová, 1990), and by qPCR (Liebig et al., 2021; Migné et al., 2022). The titer of infectious virus does not always reflect the true amount of TBEV, because it is determined by the effect of the virus on mammalian cells or organisms. During TBEV adaptation to ticks, virus variants can be selected, which are capable of long-term persistence in tick cells, and do not cause a cytopathic effect in mammalian cells (i.e., do not form plaques) or are not capable of efficient reproduction in mammalian cells (Labuda et al., 1994; Romanova et al., 2007). However, even the qPCR method has limitations and our data on higher prevalence of the strain LK-138 in ticks after trans-stadial transmission than after feeding on infected mice suggests that we may underestimate the true ticks’ IR.

In the experiment with the strain EK-328 we observed that TBEV IR in ticks was significantly affected by the time period from ticks’ feeding or molting to the analysis of the virus’s presence. According to our data, the IR in engorged larvae and nymphs was significantly higher on day 7 after feeding on infected mice than on days 30–31. Other scientists have drawn similar conclusions for the European TBEV subtype (Danielová, 1990). Perhaps this is due to physiological changes in the body of the molting tick, although, unlike insects, most organs in ticks do not undergo histolysis (Balashov, 1972). On the contrary, analysis of the effectiveness of the first trans-stadial TBEV transmission after a longer period post molting (49–84 d.p.m.) favored a higher percentage of virus-positive ticks. In the first 7–14 days after molting, the post-molting period of ticks may take place (Balashov, 1998), during which the molting processes in the tick’s body are completed, and which possibly affect the virus reproduction. Previously, controversial data have been obtained on the effect of molting on IR and virus titer (Mishaeva and Votyakov, 1978; Mishaeva and Erofeeva, 1979; Danielová, 1990). In our case, we can make comparisons between strains and tick species, when the ticks were analyzed at the same time after feeding or molting. When evaluating the efficiency of TBEV acquisition and transmission, it is necessary to consider IR obtained in the optimal period of time—shortly after feeding and later after molting.

In our work, the efficiency of the acquisition of the strain EK-328 from infected mice by *I. ricinus* and *I. persulcatus* ticks and their hybrids was generally higher than the ticks’ IRs with the strain LK-138. However, if we compare the results on the same period after feeding, then there will be no differences between two TBEV strains. We also noted that the effectiveness of the acquisition of the strain EK-328 by *I. ricinus* and *I. persulcatus* nymphs was slightly higher than by the larvae, and the difference was significant for *I. persulcatus.* It was logical to assume the influence of the feeding time of larvae and nymphs and volume of ingested TBEV-infected blood on the IR, although no differences in IR of larvae and nymphs with the strain LK-138 were observed. In our experiment, the feeding time of nymphs in all groups of ticks was the same (3 days), while the feeding time of larvae differed in tick groups: *I. ricinus* – 2 days, *I. persulcatus* – 3 days, and for hybrids it varied and averaged 2.7 ± 0.6 days. Thus, significant differences in the feeding duration of larvae and nymphs were observed only for *I. ricinus*, although no significant difference in IR of these ticks with TBEV was noted. The differences in the IR of larvae and nymphs may be associated not only with the volume of absorbed blood with the virus, but also with the sensitivity of the TBEV detection method.

Our most interesting finding was that the most effective transmission of the strain EK-328 from an infected mouse was observed in the hybrid larvae (91.7%), in which the level of virus reproduction was 2-3 log_10_GCN higher than in the parental tick species. We did not observe any differences in the number of RNA copies of various TBEV strains in *I. ricinus*. However, in *I. persulcatus* larvae, the number of RNA copies of the strain EK-328 was significantly higher than that of the strain LK-138.

It has been established that the level of trans-stadial transmission depends on many factors (strain and infectious dose of the virus, method of infection, method of TBEV detection in ticks) and can range from 0 to 100% (Naumov et al., 1980; Kurenkov et al., 1981; Danielová, 1990; Slovák et al., 2014; Migné et al., 2022). For the first time in one experiment, we showed that after trans-stadial transmission, there were differences in the IRs between two TBEV strains rather than between ticks from different experimental groups. Significantly higher values of the number of TBEV RNA copies were observed in *I. persulcatus* and *I. ricinus* ticks infected with the specific virus subtype in comparison with the non-specific subtype after the first and the second trans-stadial transmission, respectively. In hybrids, usually the highest amount of RNA of the Siberian TBEV subtype was observed during all transmission stages.

In our study, we examined the probability of TBEV detection in ticks after the second molt after feeding on uninfected mice (second trans-stadial transmission). In this case, the tick IR will depend not only on the survival of the virus, but also on the effectiveness of non-viremic TBEV transmission during the co-feeding of infected and uninfected ticks. The transmission of TBEV during co-feeding is usually effective and, depending on the host, the distance between infected and uninfected ticks and the strain of the virus, can reach 40–90% (Labuda et al., 1993; Slovák et al., 2014; Ličková et al., 2020). In our work, the IR with LK-138 in *I. ricinus* ticks after the second molt increased by almost two times compared to the first trans-stadial transmission. As a result, in *I. ricinus*, the IR with the strain LK-138 and the number of copies of this strain were much higher than those for the strain EK-328. The highest IR with the strain EK-328 was noted in *I. persulcatus*, and compared with the first trans-stadial transmission, this value increased by 3.7 times. Interestingly, the highest number of EK-328 RNA copies was preserved in hybrids even after the second trans-stadial transmission. Thus, we observed a significant increase in ticks’ IR with TBEV after the second trans-stadial transmission, apparently due to the non-viremic transmission of the virus, but only in a specific subtype–tick combination. Khasnatinov et al. (2016) showed that TBEV strains of the European and Siberian subtypes had various levels of non-viremic transmission from infected *I. ricinus* females to nymphs. In our work, we determined TBEV RNA copies in ticks using qPCR, and did not study the titer of the infectious virus in ticks, but we observed some correlation between the number of RNA copies and the IR in *I. ricinus* and *I. persulcatus*. For hybrids, this correlation was rather the opposite.

To prove the competence of hybrids as TBEV vectors, it is necessary to demonstrate not only the effective acquisition and trans-stadial transmission of the virus, but also the ability to transmit the virus to a susceptible host. In our work, we have shown the ability of hybrids to infect laboratory mice with TBEV of Siberian and European subtype, despite the small number of analyzed animals.

Thus, in this work, we analyzed the features of transmission of the Siberian and European TBEV subtypes in *I. ricinus* and *I. persulcatus* and their hybrids. Significant differences were observed in *I. ricinus* and *I. persulcatus* after the second trans-stadial transmission of TBEV, where, possibly due to non-viremic transmission, the IR of ticks increased significantly only in the case of a specific subtype-tick combination. In hybrids we observed the highest acquisition effectiveness and RNA copy numbers during Siberian subtype transmission, but the IR of hybrids in general did not differ from the parental species. Additional experiments are needed with other TBEV strains and with hybrids from another cross (female *I. ricinus* × male *I. persulcatus*) in order to study the features of transmission of different TBEV subtypes in these ticks in detail.

## Data availability statement

The original contributions presented in the study are included in the article/**Supplementary Material**. Further inquiries can be directed to the corresponding author.

## Ethics statement

The animal study was reviewed and approved by Ethics Committee of Chumakov Federal Scientific Center for Research and Development of Immune-and-Biological Products of RAS (Institute of Poliomyelitis), protocol No 040321-1.

## Author contributions

Conceptualization: OB and GK. Methodology: OB. Validation: OB and GK. Formal analysis: OB. Investigation: OB, AP and AA. Data curation: OB. Writing—original draft preparation: OB. Writing—review and editing: OB and GK. Visualization: OB and AP. Supervision: OB and GK. Project administration: OB and GK. Funding acquisition: GK. All authors contributed to the article and approved the submitted version.
