# Supplementary material for: Hybrids of Ixodes ricinus and Ixodes persulcatus ticks effectively acquire and transmit tick-borne encephalitis virus
Source: Front Cell Infect Microbiol. 2023 Jan 20;13:1104484. doi: 10.3389/fcimb.2023.1104484 (PMC9895388; doi:10.3389/fcimb.2023.1104484)
Supplement: Supplementary file 1 [file DataSheet_1.zip › Supplementary tables.DOCX]

Supplementary Material

Table S1. Specific primers used in the study

| Specific to | Name of primer | Genome locus | Oligonucleotide sequence | Primer type | Where was used |
| --- | --- | --- | --- | --- | --- |
| TBEV, all strains | TBE/Pow3’ | 3`NTR | 5’-agcgggtgtttttccgagtc-3’ | Reverse | RT |
|  | F-TBE |  | 5’-gggcggttcttgttctcc-3’ | Forward | qPCR |
|  | R-TBE |  | 5’-acacatcacctccttgtcagact-3’ | Reverse |  |
|  | TBE-probe |  | (FAM)- tgagccaccatcacccagacaca-(BHQ1) | Probe |  |
| TBEV, strain EK-328 | GTB1R | NS5 | 5’-ccattccggctctgaacttg-3’ | Reverse | RT |
|  | TBEL1 |  | 5’-tctgagggagacacacttgg-3’ | Forward | qPCR |
|  | TBER1 |  | 5’-gtgcgcctgtaaacaaagaa-3’ | Reverse |  |
|  | TBEP1 |  | (FAM)- tccttggtgcagctgttcagcc-(BHQ1) | Probe |  |
| Poliovirus | PVR1 | 3D | 5’-cgaacgtgatcctgagtgtt-3’ | Reverse | RT, qPCR |
|  | PVL1 |  | 5’-ggcagacgagaaatacccat-3’ | Forward | qPCR |
|  | PVP1 |  | (R6G)-ttgattcatgaatttccttcattggca-(BHQ1) | Probe |  |

qPCR – quantitative polymerase chain reaction; RT – reverse transcription

Table S2. Dependence of TBEV prevalence in ticks from the number of days after ticks’ feeding on infected mice and molting

| TBEV strain | Tick species | Phase | No of infected mouse | Days from the end of ticks’ feeding on infected mouse till analysis | Days from last molting (2^nd^ feeding on uninfected mice) till analysis | TBEV in ticks | |
| --- | --- | --- | --- | --- | --- | --- | --- |
|  |  |  |  |  |  | Prevalence, % [CI95%] (abs)* | Geometric mean number of RNA copies, log_10_GCN/sample ±SD * |
| TBEV acquisition from infected mice (1^#^) | | | | | | | |
| EK-328 | Hybrid | LL_en_* (1) | 1-1 | 9 | na | 91.7 [75.1-100] (27/30) | 6.6±3.05 **^i^** |
|  | *I. pers* | LL_en_ (1) | 2-1 | 9 | na | 34.1 [10.8-66.8] (7/21) **^a^** | 3.65±0.24 **^g^** |
|  |  |  | 9-2 | 31 | na | 7.2 [1.5-19.6] (3/45) **^a^** | 4.73±0.21 **^g,h^** |
|  |  | NN_en_ (1) | 5-2 | 30 | na | 50 [18.8-81.2] (3/6) | 3.88±0.21 |
|  | *I. ric* | LL_en_ (1) | 3-1 | 10 | na | 53.6 [23.7-86.4] (16/30) **^b^** | 3.59±0.09 |
|  |  |  | 11-2 | 31 | na | 4.7 [0.6-15.9] (2/45) **^b^** | 3.96±0.60 |
|  |  | NN_en_ (1) | 12-1 | 9 | na | 100 [54.3-100] (4/4) **^c^** | 3.91±0.27 |
|  |  |  | 14-1 | 9 | na | 75.0 [28.9-96.6] (3/4) **^c^** | 3.82±0.14 |
|  |  |  | 7-2 | 30 | na | 16.7 [1.1-58.2] (1/6) **^c^** | 4.04 |
| LK-138 | Hybrid | NN_en_ (1) | 4-2 | 30 | na | 25 [3.4-71.1] (1/4) | 3.57 |
|  | *I. pers* | LL_en_ (1) | 10-2 | 31 | na | 18.9 [7.7-35.7] (9/45) | 3.87±0.45 **^h^** |
|  |  | NN_en_ (1) | 6-2 | 30 | na | 16.7 [1.1-58.2] (1/6) | 3.66 |
|  | *I. ric* | LL_en_ (1) | 12-2 | 31 | na | 9.8 [2.7-23.4] (4/45) | 3.75±0.28 **^i^** |
|  |  | NN_en_ (1) | 8-2 | 30 | na | 16.7 [1.1-58.2] (1/6) | 4.06 |
| Trans-stadial TBEV transmission (I) (2-3) | | | | | | | |
| EK-328 | Hybrid | NN* (2^#^) | 1-1 | 53 | 7 | 14.3 [7.7-23.4] (15/105) | 7.90±0.36 |
|  |  | Ad (2) | 1-2 | 87 | 50 | 25 [3.4-71.1] (1/4) | 3.85 |
|  | *I. pers* | NN (2) | 2-1 | 53 | 7 | 0/21 | - |
|  |  |  | 9-2 | 115 | 76 | 11.5 [5.0-21.6] (9/78) | 3.91±0.39 |
|  |  | NN_en_ (3) | 9-2 | 168 | 129 (30) | 10.0 [0.0-42.6] (1/10) | 3.38 |
|  |  |  | 5-2 | 260 | 221 (71) | 0/6 | - |
|  |  | Ad (2) | 5-2 | 87 | 49 | 76.9 [49.1-92.5] (10/13) | 6.47±2.46 **^j^** |
|  | *I. ric* | NN (2) | 3-1 | 53 | 7 | 0.7 [0.02-4.4] (1/138) **^d^** | 4.02 |
|  |  |  | 11-2 | 115 | 84 | 8.0 [3.0-16.7] (6/81) **^d^** | 3.90±0.21 |
|  |  | NN_en_ (3) | 11-2 | 168 | 137 (32) | 21.1 [8.0-43.9] (4/19) | 3.40±0.08 |
|  |  | Ad (2) | 12-1 | 53 | 7 | 5.6 [0.0-27.7] (1/18) **^e^** | 4.36 |
|  |  |  | 14-1 | 53 | 7 | 14.3 [2.8-41.2] (2/14) **^e^** | 4.10±0.31 |
|  |  |  | 7-2 | 87 | 50 | 54.6 [28.0-78.8] (6/11) **^e^** | 4.15±0.28 |
| LK-138 | Hybrid | Ad (2) | 3-2 | 114 | 76 | 75.0 [28.9-96.6] (3/4) | 4.33±0.28 |
|  |  |  | 4-2 | 114 | 76 | 80.0 [36.0-98.0] (4/5) | 4.32±0.36 |
|  | *I. pers* | NN (2) | 10-2 | 115 | 76 | 47.7 [28.6-68.8] (30/63) | 3.75±0.34 **^k^** |
|  |  | NN_en_ (3) | 10-2 | 168 | 129 (30) | 25.0 [6.3-60.0] (2/8) **^f^** | 3.83±0.67 |
|  |  |  | 10-2 | 260 | 221 (71) | 100 [60.0-100] (5/5) **^f^** | 4.30±0.18 |
|  |  | Ad (2) | 6-2 | 114 | 75 | 45.5 [21.3-72.0] (5/11) | 3.67±0.34 **^j^** |
|  | *I. ric* | NN (2) | 12-2 | 115 | 84 | 46.9 [27.6-68.2] (28/60) | 4.24±0.18 **^k^** |
|  |  | NN_en_ (3) | 12-2 | 168 | 137 (31) | 37.5 [13.5-69.6] (3/8) | 3.96±0.47 |
|  |  | Ad (2) | 8-2 | 114 | 76 | 36.4 [15.0-64.8] (4/11) | 4.13±1.33 |
| Trans-stadial TBEV transmission (II) (4) | | | | | | | |
| EK-328 | Hybrid | Ad (4) | 1-1 | 242 | 43 (157) | 25.0 [3.4-71.1] (1/4) | 8.94 |
|  |  |  | 1-1 | 242 | 43 (138) | 16.7 [5.0-40.1] (3/18) | 5.93±2.18 |
|  | *I. pers* | Ad (4) | 2-1 | 242 | 30 (128) | 37.5 [13.5-69.6] (3/8) | 3.66±0.23 **^l^** |
|  |  |  | 2-1 | 242 | 43 (149) | 57.1 [32.6-78.7] (8/14) | 4.29±0.35 **^l,m^** |
|  |  |  | 9-2 | 218 | 35 (80) | 0/4 | - |
|  |  |  | 9-2 | 332 | 149 (194) | 14.3 [0.5-53.4] (1/7) | 3.97 |
|  | *I. ric* | Ad (4) | 3-1 | 242 | 43 (149) | 11.5 [3.2-29.8] (3/26) | 3.44±0.06 **^m,n^** |
|  |  |  | 11-2 | 218 | 35 (82) | 0/8 | - |
| LK-138 | *I. pers* | Ad (4) | 10-2 | 332 | 149 (194) | 66.7 [29.6-90.8] (4/6) | 4.20±0.10 |
|  | *I. ric* | Ad (4) | 12-2 | 218 | 33 (81) | 85.7 [46.7-99.5] (6/7) | 4.52±1.15 **^n^** |
|  |  |  | 12-2 | 260 | 5 (71) | 2/2 | 4.29±0.05 |
|  |  |  | 12-2 | 332 | 147 (195) | 1/1 | 4.24 |

na – not applicable; NN – questing nymphs; Ad – adult ticks; LL_eng_ – engorged larvae; NN_eng_ – engorged nymphs; Abset – strain Absettarov; * engorged larvae and questing nymphs were studied in pools of three, others – individually, the number of TBEV RNA copies is shown for sample (a pool or individual tick), TBEV prevalence was calculated using a Maximum likelihood estimator with the assumption of 100% test sensitivity and specificity on the EPITOOLS web platform (Sergeant, 2018); ^#^—numbers indicate different stages of the experiment according to the Fig. 1; superscript letters indicate statistically significant differences between groups according to Fisher's exact test/chi-square test (infection rate, p<0.05) or the Mann–Whitney U-test (RNA copies, p<0.05), groups with the same letter are significantly different from each other.

Table S3. Mean amount of TBEV RNA in tick suspensions at different time period post feeding on infected mice and molting. Geometric mean number of RNA copies (log_10_GCN) in the sample ±SD is presented.

| TBEV strain | Tick species | TBEV acquisition from infected mice | | | Trans-stadial transmission of TBEV (I) | | | | Trans-stadial tr. (II) | |
| --- | --- | --- | --- | --- | --- | --- | --- | --- | --- | --- |
|  |  | **LL_eng_*** (1^#^) | | **NN_eng_** (1) | **NN*** (2) | | **NN_eng_** (3) | **Ad** (2) | **Ad** (4) | |
|  |  | 9-10 d.p.f. | 30-31 d.p.f. | 30-31 d.p.f. | 7 d.p.m. | 49-84 d.p.m. | 129-137 d.p.m. (30-32 d.p.f.) | 49-84 d.p.m. | 30-43 d.p.m. | 147-149 d.p.m. |
| EK-328 | *I.ric* | 3.59±0.09 **^a^** | 3.96±0.60 | 4.04 | 4.02 | 3.90±0.21 **^d^** | 3.40±0.08 | 4.15±0.28 | 3.44±0.06 **^g,h^** | nd |
|  | *I.pers* | 3.65±0.24 **^b^** | 4.73±0.21 **^b,c^** | 3.88±0.21 | - | 3.91±0.39 | 3.38 | 6.47±2.46 **^f^** | 4.12±0.43 **^g^** | 3.97 |
|  | Hybrid | 6.6±3.05 **^a^** | nd | nd | 7.90±0.36 | nd | nd | 3.85 | 5.41±2.06 **^g^** | nd |
| LK-138 | *I.ric* | nd | 3.75±0.28 | 4.06 | nd | 4.24±0.18 **^d,e^** | 3.96±0.47 | 4.13±1.33 | 4.52±1.15 **^h^** | 4.24 |
|  | *I.pers* | nd | 3.87±0.45 **^c^** | 3.66 | nd | 3.75±0.34 **^e^** | 3.83±0.67 | 3.67±0.34 **^f^** | nd | 4.20±0.10 |
|  | Hybrid | nd | nd | 3.57 | nd | nd | nd | 4.32±0.30 | nd | nd |

NN—questing nymphs; Ad—adult ticks; LL_eng_—engorged larvae; NN_eng_—engorged nymphs; GCN—genome copy number; SD—standard deviation; d.p.f.—days post feeding; d.p.m.—days post molting; *—engorged larvae and questing nymphs were studied in pools of three; ^#^—numbers indicate different stages of the experiment according to the Fig. 1; superscript letters indicate statistically significant differences between groups according to the Mann–Whitney U-test (2 groups) or Kruskal-Wallis H-test (3 groups) (p<0.05), groups with the same letter are significantly different from each other
